# Some Impressions of the 1914 British Pharmacopœia

**Published:** 1914-12

**Authors:** Oliver C. M. Davis

**Affiliations:** Lecturer on Materia Medica in the University of Bristol


### SOME IMPRESSIONS OF THE 1914 BRITISH PHARMACOPŒIA.

BY

Oliver C. M. Davis, D.Sc.,

*Lecturer on Materia Medica in the University of Bristol.*

---

The present contribution is a somewhat modified account of a paper brought before the Bristol Medico-Chirurgical Society by the writer on November 11th.

No attempt has been made to treat the matter in minute detail, and only certain outstanding alterations have been dealt with and criticised, and special reference has been made to those subjects which concern medical practitioners.

One of the most striking features of the new work is the introduction of the metric system of measuring and weighing drugs for the various preparations without giving at the same time directions involving the use of the Imperial System. The doses, however, are given in both systems, which is fortunate for many people. The "standard litre" is taken as the volume of one kilogram of water measured at 4° C.; the volume of 1 gm. of water also measured at 4° C. is termed a "millilitre" or "mil," and this is sub-divided into the "decimil" and "centimil."

During recent years there have been many discussions concerning the relative efficacy of pure chemical compounds and indefinite preparations in medical practice. There can be little doubt that the medicinal values of drugs were originally determined by observing the effects of crude natural products when administered internally; as a natural sequence to such discoveries it became obvious that in many cases a better result could be obtained by giving the soluble constituents of a drug without the insoluble inert matter, and in this way infusions, decoctions, extracts, and later tinctures, came into general use. At a comparatively recent period such a system was purely empirical, as neither the active principles of drugs nor their physiological action had been investigated.

With the rise of organic chemistry and pharmacology during the last century numerous organic compounds were isolated in a state of purity from crude drugs, and in many cases their action has been investigated by experimental pharmacologists. Such researches have laid the foundations of a rational system of pharmacology, and the further introduction of very numerous synthetic drugs has greatly strengthened this system. It must be freely admitted that such a system is at present very far from perfect, and very greatly in need of improvement, but it is surely the only one that can eventually put clinical pharmacology on an absolutely firm basis.

One fully recognises that with our present state of knowledge we are compelled to retain a considerable number of very valuable preparations, such as Tinct. Nucis Vom., Tinct. Opii, etc., etc. ; in fact, there is no reason why such well-established galenicals should ever fall into disuse, but in the writer's opinion too many new preparations of a similar type have been introduced. For example, it seems superfluous to include a vinegar, oxymel, syrup and tincture prepared from Urginea Indica, in addition to the already existing corresponding preparations made from Urginea Scilla.

The introduction of preparations of this type into such an important work as the *British Pharmacopœia* is not calculated to advance the science of pharmacology. At the same time, it is inspiring to see that the vinegar, plaster, ointment and tincture of Cantharides have been replaced by corresponding preparations made from the active principle Cantharidin.

Several synthetic preparations are included for the first time, and as mentioned elsewhere their names have been modified. Thus :—

| *New name.* | *Name of Patent Product of same composition.* |
|---|---|
| Barbitonum. | Veronal. |
| Benzamine Lactas. | Eucaine Lactate. |
| Chloral Formamidum. | Chloralamide. |
| Diamorphine Hydrochloride. | Heroin Hydrochloride. |
| Hexamina. | Urotropin. |
| Methyl Sulphonal. | Trional. |
| Phenolphthalein. | Numerous names. |

With regard to the abbreviated nomenclature adopted for some of the latter class of drugs criticism is necessary. In the case of such a compound as Benzoyl-vinyl-diacetonalkamine lactate, the full name would be rather cumbersome, and the more convenient " Benzamine Lactate " appears to be justified. But it is extremely doubtful whether the same justification exists in the case of Barbitonum, Hexamine, and Diamorphine Hydrochloride, all three of which might have well retained their constitutional names, which are respectively Diethyl-malonyl-urea, Hexamethylene-tetramine and Diacetyl-morphine-hydrochloride. This scientific method is actually adopted in the case of Trional, which being a methyl substitution product of Sulphonal is called Methyl-sulphonal.

Other important synthetic drugs have been included, and it is to be regretted that the number is not greater.

A hypodermic injection containing 0.75 per cent. of Strychnine Hydrochloride has been included for the first time.

Quite a large number of preparations have been omitted, and several of these omissions strike one as being unsatisfactory, notably Ferri Arsenas, Ferri Phosphas, Sodii Sulphocarbolas, Zinci Sulphocarbolas, and Tinct. Jaborandi; but of these Ferri Phosph. has been replaced by a saccharated preparation, and the scarcity of jaborandi leaves may account for the deletion of the tincture.

One uncalled-for class of preparations, the Concentrated Liquors, has been deservedly removed, and this withdrawal takes away a conspicuous blot from a scientific work; if a clinician orders infusions or decoctions they should be freshly made, or there is no point in using them. The Concentrated Liquors presumably took the place of concentrated infusions and decoctions, which, though convenient for the pharmacist, do not possess the same properties as recent preparations.

With regard to those preparations which have had their concentrations altered, both favourable and unfavourable criticisms must be made. Certain of these alterations have been made in order to bring about an International Conformity, whereas others have been made for less obvious reasons. Such changes render it urgently necessary for physicians to re-learn the new strengths and doses, and this is a most unsatisfactory state of things. The clinician has so many responsibilities associated with his work, that it becomes almost criminal to burden him by compelling him to unlearn facts just to satisfy the whims of a few people.

Thus an important preparation, Tr. Strophanthi, is four times the strength of the old preparation, and the dose has been changed from 5-15 mins. to 2-5 mins. Tr. Opii now contains 1 per cent. morphia instead of 0.75 per cent., and Tr. Nucis Vom. is half the strength of the old preparation! One mercurial ointment, Ung. Hydrarg. Am., has been reduced from 10 per cent. to 5 per cent.; another, Ung. Hydrarg. Subchlor, has been increased from 10 per cent. to 20 per cent. The former change has been brought about in deference to certain eminent dermatologists, and needs no comment. The increased strength of Calomel Ointment is doubtless due to the fact that it is largely used by a certain section of the community as a prophylactic against the unhappy protozoon Treponema Pallidum, which has to comply with the law of mass action.

It is pleasing to notice that the hypodermic injections of Cocaine and Morphine have both been reduced to one-half their former strength. This is most satisfactory, as they were formerly too concentrated to be accurately measured by the methods adopted clinically, and now there should be far less chance of error arising.

The introduction of the Metric System is responsible for at least two slight changes in strength: Syr. Chloral Hydrat. formerly contained 10 grs. in each fluid drachm, and Liq. Hydrarg. Perchlor. $\frac{1}{16}$ gr. in one fluid drachm, or approximately 0.115 grs. in 110 mins. The former now contains 200 gm. in 1,000 millilitres or 10.9 grs. in a fluid drachm, and the latter now contains 1 gm. in 1,000 millilitres or $\frac{1}{10}$ gr. in 110 minims.

The method adopted for the representation of formulæ of organic compounds calls for serious criticism in many instances. It must be realised that the *Pharmacopœia* is used as a text-book by many students, and the crude method adopted for organic formulæ does not conduce to an intelligent comprehension of chemistry or pharmacology. To take a few examples, Phenol or Carbolic Acid is represented as $C_6H_6O$. As a purely empirical formula this may be justified, but it conveys no idea whatever either of the chemical constitution or the probable physiological action. If the formula were written $C_6H_5OH$ it would reveal the fact that phenol is the mono-hydroxyl substitution product of benzene, and one would expect it to give the chemical reactions of both the phenyl and hydroxyl group. One would also surmise that the hydroxyl group could anchor the phenyl group to living protoplasm and thus enable it to bring about a physiological action.

The formula $C_8H_9NO$ given for Acetanilide is another very striking example of the ludicrous method adopted in most cases. On reference to the latest edition of Richters' *Dictionary of Carbon Compounds* it will be found that no less than sixty-two compounds lay claim to the formula $C_8H_9NO$, whereas only one compound, Acetanilide, is represented by the constitutional formula $C_6H_5NH.COCH_3$.

It is curious to notice that a logical method is adopted in the case of Picric Acid, which is represented as $C_6H_2(NO_3)_3$ OH !

There is one direction in which improvement was desirable, and still is so. It would be an enormous benefit to medical men if the concentrations of tinctures, spirits, liquors, syrups, and so forth, could be so adjusted that they possessed the same dose, as the variety of doses now obtaining is puzzling even to a man with a good memory, and hopeless to many ; obviously certain exceptions would have to be made, but on the whole there is no serious objection to a greater uniformity of dose than now exists.

The appendix contains much valuable information, especially concerning analytical chemistry, and is in advance of previous appendices.

